# Building like a Coral—Parallelized, Multiscale Biofabrication

**DOI:** 10.1002/adma.202520519

**Published:** 2026-02-25

**Authors:** Asma Rehman, Marta Peña Fernández, Kristina K. Beck, Gavin L. Foster, Sebastian J. Hennige, Uwe Wolfram

**Affiliations:** ^1^ Institute of Materials Science and Engineering Clausthal University of Technology Clausthal‐Zellerfeld Germany; ^2^ School of Engineering and Physical Sciences Heriot‐Watt University Edinburgh UK; ^3^ Changing Oceans Research Group School of GeoSciences University of Edinburgh Edinburgh UK; ^4^ School of Ocean and Earth Science University of Southampton Southampton UK

**Keywords:** aragonite, biomineralization, calcification, cold‐water corals, scleractinian corals, structural materials, sustainable manufacturing

## Abstract

Visible from space or residing in the depths of the ocean, scleractinian corals engineer vast ecosystems supporting high biodiversity and providing essential ecosystem services. By creating these ecosystems, corals address significant challenges in material science, generating skeletal materials that are stiff, strong, and inherently circular—even in conditions where energy and building resources can be scarce or energetically expensive to synthesize. Understanding coral skeletal materials has progressed due to their exceptional mechanical properties, potential biocompatibility, and, in case of cold‐water corals, their ability to be synthesized in darkness, at low temperature, and with limited energy resources. These natural, sustainable processes offer inspiring blueprints for the development of transformative new materials, which may drive radical innovations across biomedical and engineering applications. In this perspective, we synthesize the current state of knowledge on the biomineralization process of corals, including the two prevailing viewpoints—biologically controlled vs. physicochemical controlled biomineralization. We then recast coral growth as a multiscale, parallelized biofabrication process, that can catalyse the development of next‐generation materials technologies. These insights outline pathways to sustainable, self‐organising, and energy‐efficient manufacturing with broad relevance to structural materials, biomaterials, and regenerative engineering. Ultimately, we strive to answer: “How to build like a coral?”

## Graphical abstract

Corals build stiff, strong, and inherently circular skeletal materials under resource‐ and energy‐limited conditions—offering blueprints for transformative materials. We synthesize the current understanding of coral biomineralization and reframe coral growth as a multiscale, parallelized biofabrication process. We outline pathways to sustainable, self‐organising, energy‐efficient manufacturing with relevance to structural materials, biomaterials, and regenerative engineering.

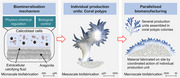

## 1 Introduction

Scleractinian, or stony corals, are ecologically significant as they are the primary architects of coral reefs [1, 2], which are biodiverse marine ecosystems. These reef‐building corals, existing in tropical, subtropical, and cold‐water habitats, are the only anthozoans capable of producing an external mineral skeleton during their settlement and metamorphosis [3]. Coral skeletal material exhibits exceptional precision, structural organization, and impressive mechanical properties, durability, and resilience against fractures, making it valuable for various applications in a range of sectors, from biomedical to engineering [4].

Tropical corals thrive near their maximum thermal tolerance limit and most reef building species live in symbiosis with photosynthetic dinoflagellates of the family Symbiodiniaceae (*zooxanthellae, Symbiodinium*) [5]. These endosymbionts reside within the gastrodermal (endodermal‐derived) cells and provide an energy source that supports coral metabolism and skeletal formation mechanisms, processes that would occur at lower rate without them [6, 7]. In contrast, deep‐sea or cold‐water corals (CWC) reside at depths of 40–4000 m [8], i.e. in dark and colder waters [9]. Lacking zooxanthellae, they rely solely on food particles from surface waters and zooplankton, a supply that diminishes with increasing depth [10, 11]. Despite these challenges, CWCs enable vibrant marine ecosystems [9, 12].

Corals are among the most rapidly mineralizing organisms in marine environments [13, 14]. They produce ≈10^12^ kg of calcium carbonate (CaCO_3_) annually [15] and construct the largest biogenic structures on Earth [16]. Corals use Ca^2+^ and CO_3_^2−^ ions from surrounding seawater to fabricate skeletal material in the interstitial space between the polyp base and the existing skeleton [17]. Although under tight biological control, this process is significantly influenced by environmental conditions [18, 19, 20]. The rapid accretion of CaCO_3_ occurs under favorable conditions [21], such as optimal temperatures, adequate light (although there is evidence that they grow during the night [22]), nutrient availability, and high CaCO_3_ saturation in case of tropical corals [23, 24] and, stable, cooler temperatures and sufficient supply of particulate organic matter in case of CWCs [25]. However, the mechanisms underlying coral CaCO_3_ production remain poorly understood, leading to the existence of a polarized view of biomineralization as either a biologically or physicochemically controlled mineralization processes [17, 20, 26, 27].

The biominerals forming the coral skeleton consist of crystalline materials with nano‐ or micro‐scale features and an organic framework [28]. Recent research indicates that proteins [29, 30, 31], glycans [32], and lipids [33] are crucial in shaping the microstructure and interfacial properties of these biominerals. Glycans within the organic matrix modulate mineral‐phase growth by mediating mineral–matrix interactions [34], whereas lipids influence crystal growth and arrangement [35]. As a result these components affect the unique physical [36, 37] and mechanical [14, 38] properties of the coral skeletal material. Coral acid‐rich proteins (CARPs), also referred to as skeletal aspartic acid‐rich proteins (SAARPs) [39], are thought to be crucial in nucleation [15, 31, 40] and in the selection of CaCO_3_ polymorphs that corals utilize to construct their skeletons [31]. Among these polymorphs, aragonite is the predominant form in mature skeletons, and CARPs significantly influence the development of aragonite skeletons [30, 31, 41]. The organic phase may alter the fundamental characteristics of mineral crystals through incorporation during crystal growth [30]. The spatial arrangement of organic constituents relative to the mineral phase, as well as the material architecture, appears crucial for the formation and performance of these biocomposites [42]. However, questions remain where these components are located and how they impact skeletal properties. Well‐designed interfaces are known to connect disparate materials, enhancing elasticity and durability in other natural materials, such as mollusc shells, bones, and insect exoskeletons [43]. How these are precisely realized in corals remains not fully understood.

**FIGURE 1:**
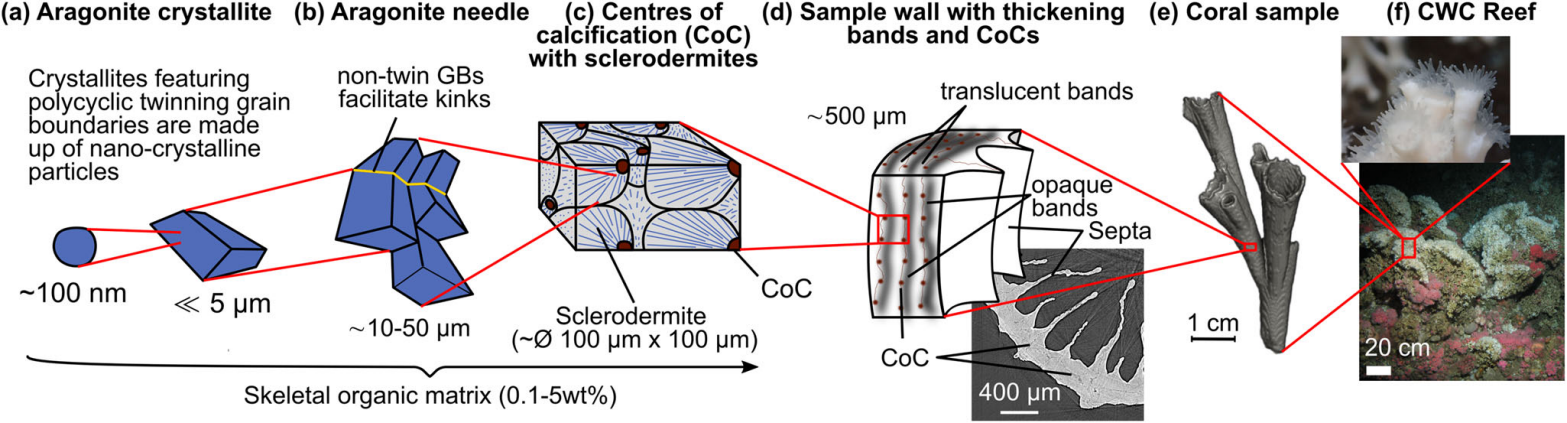
Material architecture of a branching cold‐water coral (CWC) from aragonite crystallite to reef. CWCs employ aragonite crystallites (a) to form needles (b) which are used to construct a polycrystalline skeletal matrix, incorporating an organic‐calcium carbonate ‘cement’ within the 3D framework [40, 44, 45, 46, 47] (c). Sclerodermites (c), whose existence is debated [47, 48], make up structure walls (d) which form individual corallites (e) that ultimately arrange in a branching pattern to form entire reef systems (f). The synchrotron radiation micro‐computed tomography image in (d) shows centers of calcification (CoCs) and septa in a *Lophelia Pertusa* sample obtained by Hennige et al. [44] Septa are the vertical partitions that extend inward from the theca wall and contain CoCs of calcium carbonate [48, 49], from which the aragonite crystals protrude (Section 5). The magnification in image (f) depicts *L. pertusa*. Images (a–e) are composed from several studies [40, 47, 48, 50, 51, 52] and our research [44, 53, 54]. Earlier versions of the image were used in [44, 53]. The figure illustrates the material architecture of cold‑water coral *L. pertusa*  due to its relevance for our own findings. Tropical (shallow‑water) corals share an analogous material hierarchical and modular organization [55, 56, 57] so that the principles illustrated are broadly applicable.

By combining mineral and organic phases, corals produce composite materials with properties that can equal or exceed those of synthetic materials or engineering analogues. For instance, the skeletal material synthesized by CWCs exhibits a compressive strength of 462 MPa [53] and stiffness ranging from 45 to 77 GPa [14, 44, 53, 58, 59], making it ten times stronger than concrete [60] and twice as strong as ultrahigh‐performance fiber‐reinforced concrete [61]. Additionally, coral materials demonstrate excellent energy dissipation and fracture resistance [62, 63], attributed to their unique material architecture [44] (Figure 1). Coral skeletons comprise aragonite needles typically 10–50 µm in length [47]. These aragonite needles consist of crystallites, or meso‐crystals, approximately 5 µm in size [47] which, in turn, are made up of 100–400 nm sized particles [64, 65].

These crystals are integrated with an organic matrix consisting of 0.1–5.0 wt.% organic carbon [40, 46] and a sub‐micrometer porosity of approximately 3.9% [45]. These aragonite needles radiate from centers of calcification (CoCs) [47, 53], also known as rapid accretion deposits (RADs) or early mineralization zones (EMZ), which are rich in certain trace elements (e.g. Mg) [64, 66], have distinct stable isotopes [67, 68], and high concentration of organic matrices [49, 69]. Such environments serve as nucleation centers for crystal formation, accomplishing coral extension. CoCs are essential for mineralization, structural integrity, and resilience of corals [20].

As aragonite needles extend, they establish a framework for the sequential deposition of new CaCO_3_ layers. Each layer forms as the polyps expand and deposit CaCO_3_, contributing to the skeleton's bulk. The thickness and density of these layers can vary based on various factors such as food and nutrient availability, seawater temperature, seawater pH or aragonite saturation, light levels and currents, all influencing the calcification rate [70]. The layering differs significantly among coral types. For example, tropical coral colonies with a massive morphology exhibit a stable, rounded structure that optimizes surface area on the seafloor. This concentric layering creates a solid framework, allowing continuous growth while providing stability [71, 72]. Conversely, branched corals grow vertically, maximizing access to resources such as sunlight (tropical corals) and food (tropical and deep sea corals [73]). However, little is known about the layering in branched corals, which occurs at branch tips in response to environmental conditions, often leading to less coherent structures [74]. Typically, branched corals display a density gradient, with thicker layers at the base for structural support [74]. Massive colonies are better equipped to withstand mechanical stresses due to their geometry [12], while branched colonies must adapt their arrangements, such as employing vertical branches for larger colonies and inclined branches in high‐energy environments to reduce stress from weight and hydraulic forces [75].

Coral biomineralization, along with the resilience of coral skeletons to biological and environmental factors, is fundamental to reef ecosystem success [76]. The anthropogenic increase in atmospheric CO_2_ is driving ocean acidification [77, 78], which poses significant risks to both tropical and CWC reefs by disrupting the delicate balance between skeletal growth and dissolution processes [79]. Ocean acidification may reduce growth rates in living corals [80] and contribute to the disintegration of dead coral frameworks, skeletal material that is no longer covered with live tissue [81]. As in terrestrial or freshwater ecosystems, this dead material is essential for maintaining various ecosystem functions, including habitat formation and carbon storage [12]. For example, CWC reef habitats and their supporting biodiversity depend heavily on dead coral skeletal material. Consequently, ocean acidification presents a more severe threat to CWC reefs than to tropical coral reefs [44]. Furthermore, ocean acidification will raise the aragonite saturation horizon, the critical level below which aragonite becomes undersaturated. Thus, by the end of this century, it is expected that approximately 70% of CWC reefs will be located below this threshold [44, 82], inhabiting waters less capable of sustaining stable aragonite mineral formation [83]. This will likely cause dissolution of exposed skeletal structures, potentially leading to the collapse of entire reef habitats [44, 53]. A better understanding of the biomineralization processes responsible for creating both skeletal material and the bulk framework of corals may provide insights into their future in a changing ocean environment, ultimately informing conservation efforts.

The mechanical properties of coral skeletons are exceptional, rooted in a unique and sophisticated biomineralization processes. Despite recent studies describing these processes, gaps remain in understanding how they contribute to the construction of robust coral frameworks with remarkable mechanical properties. These materials offer exciting potential for developing biocompatible, high‐performance applications [84, 85]. Researchers have endeavored to replicate coral skeletal materials in vitro through coral growth [86, 87] or various fabrication techniques [88, 89], including 3D printing [90, 91, 92], which are discussed in detail in section 6. However, the processes by which corals construct frameworks from individual crystals to whole reefs as well as how these processes could be translated into biomedical or broader technical application is incomplete. In particular, the dichotomy of thought regarding coral mineralization (biologically vs. physicochemically controlled coral calcification, Section 3.3) has created an artificial separation in knowledge. Here we hypothesize that corals utilize both biological and physicochemical pathways to synthesize their skeletons, thereby bridging these two viewpoints. The interplay between these processes enhances the efficiency of CaCO_3_ mineralization, producing a material that is both mechanically robust and biologically active.

This review aims to investigate how corals construct their skeletal material and develop significant structures over relatively short time scales. To address this, we will (i) provide a brief overview of the life cycle and anatomy of corals; (ii) explore the biotic and abiotic pathways involved in mineralization, focusing on potential interactions between organic matrices and CaCO_3_; (iii) examine the mechanisms of bulk skeletal material production at macroscopic length scales; and (iv) explore how these processes interconnect to form a multiscale, parallelized biotechnological framework for manufacturing these biomaterials.

## 2 Coral Biology

### 2.1 The Life Cycle of a Coral

Adult corals reproduce sexually through two primary methods: broadcast spawning and brooding [93]. In broadcast spawning, corals release sperm and eggs into the surrounding water, enabling external fertilization [94]. In contrast, brooding corals fertilize their eggs inside female polyps and retain the developing larvae until they mature [95]. Both reproduction mechanisms include a free‐swimming larval stage followed by a predominantly benthic stage [96, 97]. Once the planula larva has settled on a hard substrate, it undergoes metamorphosis into a primary polyp, accompanied by significant physiological and morphological transformations. At this stage, the settled larvae also begin the formation of an exoskeleton [98, 99].

In addition to sexual reproduction, coral polyps are able to reproduce asexually through budding, creating a new polyp that is a clone of the parent polyp [100, 101]. Living buds emerge around the outer edges of “parent” polyps, extending the soft tissue coverage and representing a new unit able to generate mineralized skeleton [102]. This generates repetitive calcification units with distinct structural characteristics [54]. Although the precise mechanism, time and need of polyp expansion by budding remains elusive, the successive budding results in the formation and enlargement of the colony [103].

Another method of asexual reproduction in corals is fragmentation [104], which occurs when pieces of a coral colony break off due to physical disturbances such as storms, wave action, or predation [105, 106]. Once these fragments detach, they can settle and reattach through calcification onto suitable substrates and begin to grow, developing new polyps that may ultimately form a new colony [104]. This asexual reproduction process enables corals to rapidly colonize new areas and recover from disturbances, thereby enhancing their survival in various environmental conditions [107, 108].

### 2.2 Coral Anatomy

**FIGURE 2:**
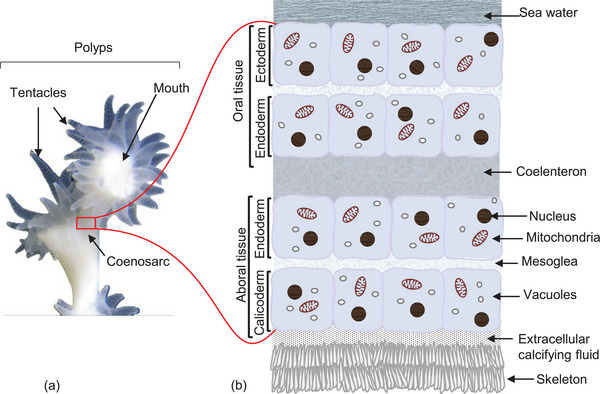
Coral anatomy: (a) illustrates schematically the microanatomy of polyps and (b) shows skeletal and soft tissue structures of colonial corals; image is adopted with permission from Drake et al. [3]. The figure uses the cold‐water coral *L. pertusa* as an example and therefore Zooxanthellae are not shown here. These crucial symbionts for energy supply in many tropical corals would reside in the oral endoderm. The cellular layers are, however, similar with regards to generating the calcified skeleton.

Corals maintain their intricate lifestyles and interact within the marine environment through a range of anatomical structures that facilitate the necessary biological functions. Figure 2a illustrates the macro‐anatomy of a coral polyp. The tissue interfacing surrounding waters is composed of two tissue layers: an outer layer (the ectoderm) and an inner layer (the endoderm). These layers are separated by a non‐cellular gelatinous layer referred to as mesoglea (Figure 2b).

The endoderm faces the coelenteron which is the primary tissue for digestion and circulation. This structure is consistent throughout the coral. While the oral tissue faces the seawater for feeding and respiration, the aboral tissue faces the exoskeleton, providing support and connection to the skeletal structure [109]. In case of zooxanthellate corals, symbiotic microalgae live within the cells of the oral tissue enabling them to utilize photosynthesis as an energy source. Given their location, these algae do not directly affect the calcification process [17]. However, they affect the growth rate of the coral host by providing energy and fulfilling its metabolic needs [110, 111].

The aboral ectoderm is again made up of two layers: an endodermal layer facing the coelenteron and the calicoderm containing calicoblasts (Figure 2b). Calicoblasts are the cells responsible for secreting the mineralized matrix [112]. The genetic profile of calicoblasts is distinct from that of other cell types within the same polyp [113, 114]. Gene expression in these cells is specifically associated with carbonate formation and transport, including other ion transporters such as those for Na^+^/HCO_3_^−^ [113, 115]. Calicoblasts were not observed in swimming larvae but they were observed in high abundance in settled polyps [113], highlighting the substantial role of calicoblasts in skeleton formation.

In case of reproduction by budding, the buds arise from, and remain connected to, the parent polyp by the coenosarc [103]. This tissue covers the skeleton between individual polyps. The coenosarc enables nutrient transport between polyps [116], increases the skeletogenic surface area [117] as it contains calicoblasts, and facilitates coral‐colonial integration of new buds [118]. To the best of our knowledge, no definite timeframe for budding has been established. Different coral species exhibit varying budding intervals, ranging from days to months. For example, Gilis et al. [119] reported the initiation of budding in *Pocillopora damicornis* polyps that had settled for approximately 10 days, while Lin et al. [120] noted the first budding in *Galaxea fascicularis* after about 7 months of settlement. Heran et al. [99] reported no polyp budding over a 3.1‐year observation period for the CWC *Caryophyllia huinayensis*. However, this is a solitary CWC species which may be why budding does not occur that frequently. While it has been established that CWC can grow at rates comparable to those of tropical corals [121], skeletal mass tends to increase at a slower pace compared to their tropical relatives [122, 123, 124] because CWC may prioritize faster soft tissue growth over rapid skeletal formation. Guo et al. [125] identified fibroblast growth factors and their corresponding receptors as potential proteins involved in budding among different reef‐building corals. However, the specific mechanisms of the fibroblast growth factor signaling pathway in coral growth remain unclear.

### 2.3 Skeletal Structure

**FIGURE 3:**
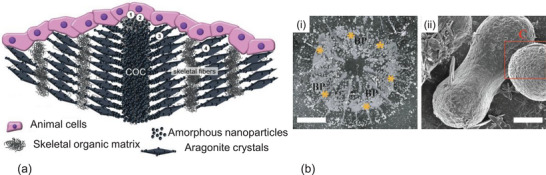
Mineral formation in corals: (a) shows a working model of coral biomineralization and how structures are built [56]. Step 1, secretion of the skeletal organic matrix by calicoblasts. Step 2, deposition of magnesium‐rich amorphous calcium carbonate nanoparticles mediated by the organic matrix. Steps 1 and 2 might happen simultaneously. Step 3, growth of acicular aragonite crystals. Step 4, formation of the skeletal fibers through the “layered model” as proposed by Stolarski [48]. Image taken from van Euw et al. [56]. (b) primary polyps of *Stylophora pistillata*: (i) after removal of the tissue; scale bars: 300 µm, (ii) pristine dumbbells (the red square highlights that pristine dumbbells are made up by nano‐granules, see Neder et al. [15] for details); scale bars: 2 µm. Image adopted from van Euw et al. [56] with permission and Neder et al. [15] under terms of the CC‐BY license.

Each coral polyp resides in a corallite, which is a tubular, hollow structure characterized by radial septa extending inward (Figure 1). The shape of the corallite is taxon‐specific and genetically controlled [126, 127]. Each septum consists of two key skeletal features (Figure 1): (i) CoC and (ii) fasciculi, or thickening deposits (TDs) [48]. The CoC comprise organic molecules and sub‐micrometer sized mineral granules [49, 128] (Figure 3), while the TDs are made up of larger acicular aragonite needles, sometimes referred to as aragonite crystal fibers, which measure approximately 10 µm in diameter and 50 µm in length [47, 129].

These aragonite needles grow along their (001) axes [47] and radiate outward in bundles from the CoC, forming more complex structures called sclerodermites or trabeculae, which constitute the framework of the bulk coral skeleton [47, 130]. Sclerodermites range from 100 to 250 µm in length and approximately 100 µm in diameter [47], displaying a spherulitic structure in which aragonite needles radiate from a common center [130]. Each acicular aragonite needle comprises smaller subunits called crystallites, which are less than 5 µm in size and slightly elongated along the (001) direction (Figure 1). The adjacent aragonite crystalline domains exhibit minor orientation differences within a range of 30°–40° [47], which may contribute to the overall strength of the skeleton [131].

Complementing these observations, in some coral species, further microstructural complexity has been identified between CoC and aragonite needles. Holcomb et al. [129] described the potential formation of a layer of granular material, referred to as nano crystals, with a diameter of less than 1 µm, situated between the CoC and the aragonite needles. These aragonite needles radiate outward from regions of this granular material, exhibiting a pattern of gradual thickening. Similar patterns of structural variation are also evident in some CWC species [132, 133], which feature a transitional zone known as “CoC zone‐like” [134] or “ion‐spots” [135] situated between the CoC and TDs. While these ion‐spots are geochemically similar to the CoC, they lack distinct morphology, indicating regions where chemical conditions favor mineralization without yet forming defined crystal structures [135]. Rich in ions, these CoC zone‐like spots likely results from the contributions of skeletal structures from both the CoC and the thicker theca wall and may contribute to the gradual thickening of aragonite needles [136].

## 3 Coral Biomineralization

**FIGURE 4:**
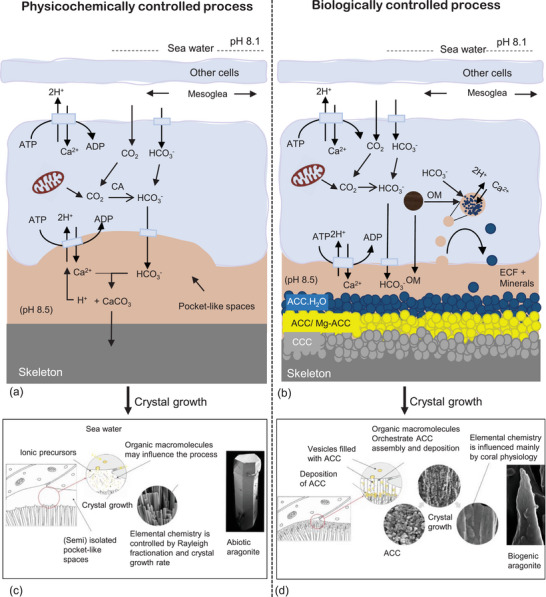
Mineralization models: (a) Schematic representation of the physicochemical model of calcification in corals, illustrating the pathway of ionic species and CO_2_ transport through calicoblastic cells, image adopted from Allemand et al. [27]; (b) schematic representation of the biological model of calcification in corals, illustrating the formation and expulsion of intracellular vesicles and nucleation of crystalline calcium carbonate (CCC) in extracellular calcifying fluid, image adopted from Sun et al. [144]; (c,d) crystal growth by physicochemically dominated process and biologically dominated process respectively, adopted with permission from Drake et al. [3].

The mechanism through which corals build and structure their CaCO_3_ skeleton has been studied extensively [137, 138, 139]. There are two prevailing viewpoints for biomineralization:

- Physicochemical calcification which advocates a physicochemically dominated process in which the coral skeleton precipitates freely in pocket shape spaces. These spaces are located between the skeleton and calicoderm (Figure 2b and Figure 4a) and contain calcification fluid that is more oversaturated with respect to aragonite than the surrounding seawater. Both the spaces as well as the calcification fluid are subject to intricate metabolic regulation by the polyp [17, 140, 141].
- Biologically controlled calcification which advocates for a biologically regulated mechanism in which the organic matrix produced by the organism is crucial for initiating and controlling the calcification process [46, 56, 142]. Here as well, the process is subject to intricate metabolic regulation by the polyp.

In general, coral calcification requires the involvement of at least two ions that react to produce CaCO_3_ as shown in reaction (1) [20], where the carbonate is directly precipitated from seawater.

(1) $$Ca^{2+}+{\mathrm{CO}}_{3}^{2-}\;\Leftrightarrow \;\mathrm{CaC}O_{3}$$

Carbonate ions (CO_3_^2−^) can be derived from bicarbonate ions (HCO_3_^−^) produced by the hydration of CO_2_ and subsequently react with Ca^2+^ to form CaCO_3_ (reaction 2). The reaction of CO_2_ with water [27] is catalyzed by the enzyme carbonic anhydrase (CA) and Ca^2+^ binds to CO_3_^2−^ ions by the following reaction (3) [27, 141]

(2) $$Ca^{2+}+{\mathrm{HCO}}_{3}^{-}\;\Leftrightarrow \;\mathrm{CaC}O_{3}+H^{+}$$

(3) $$CO_{2}+Ca^{2+}+H_{2}O\;\Leftrightarrow \;\mathrm{CaC}O_{3}+2H^{+}$$

Two H^+^ ions produced in this reaction are removed from the calcification site by Ca^2+^‐ATPase in exchange for Ca^+2^ ions across the calicoblastic cell membrane [27, 143].

Moreover, CO_3_^2−^ can also be derived from HCO_3_^−^ already available in seawater and can bind with Ca^2+^ resulting in reaction (4) [143, 145, 146].

(4) $$Ca^{2+}+2{\mathrm{HCO}}_{3}^{-}\;\Leftrightarrow \;\mathrm{CaC}O_{3}+H_{2}O+CO_{2}$$

### 3.1 Physicochemical Coral Calcification

The physicochemical model of coral calcification [17, 141, 146, 147] (Figure 4a) presumes that the extracellular calcifying fluid is ultimately derived from seawater but is modified by the coral, notably by altering its pH [146] and manipulating the dissolved inorganic carbon (DIC) [26] present in the fluid. It is thought that key to this process is Ca^2+–^ATPase, which functions as a Ca^2+^/H^+^ ion exchanger. It actively transports Ca^2+^ into the extracellular calcifying fluid in exchange for two H^+^ ions to maintain charge neutrality. This exchange ultimately increases the Ca^2+^ ion concentration and pH within the extracellular calcifying fluid [141]. The transport mechanism is powered by the energy released during the hydrolysis of adenosine triphosphate (ATP) [148]. During cellular metabolic processes, ATP is hydrolyzed into adenosine diphosphate (ADP) and, in some cases, further into adenosine monophosphate (AMP) [149], releasing energy utilized for various cellular functions [149].

In seawater, DIC exists in several chemically interconvertible species (CO_2_, HCO_3_^−^, and CO_3_^2−^), and their equilibrium is dependent on seawater pH [150, 151]. The elevated pH in the extracellular calcifying fluid shifts the equilibrium toward HCO_3_^−^ and CO_3_^2−^, making these species available to react with Ca^2+^ ions. This shift concurrently reduces the concentration of aqueous CO_2_ in the calcifying fluid, prompting diffusion of CO_2_ across the calicoblastic membrane [141]. According to McCulloch et al. [78] this biologically controlled pH up‐regulation is a critical adaptive mechanism in corals that supports the formation of CaCO_3_ even under changing oceanic conditions. Their IpHRAC model (Internal pH Regulation of the Calcifying fluid with Abiotic Calcification) combines internal pH control with abiotic precipitation kinetics to show how corals can maintain or even increase calcification rates under elevated CO_2_ conditions by sustaining high saturation states at the site of calcification. In addition to CO_2_ sourced from seawater, the carbon utilized for CaCO_3_ formation also arises from mitochondrial activity during cellular respiration [152]. HCO_3_^−^ can also be increased in the calcifying fluid by direct transport via a bicarbonate ion transporter, with those belonging to the SLC4 family being important to coral biomineral formation [153, 154].

The physicochemical model further posits that rapid calcification occurs within pockets formed between the calicoderm and the mineralized skeleton (Figure 4c). These pockets, which are approximately 20×20×10 µm in size [155], are created by the organism itself. To facilitate their formation, the coral lifts the calicoblastic layer away from the surface of the skeleton [156, 157]. Barnes [138] suggested that this action helps establish an environment favorable for crystal growth. This concept was later expanded upon by Cohen and McConnaughey [17], who indicated that the increase in osmotic pressure within the calcifying space forces the ectoderm away from the skeleton's surface, thereby creating a space conducive to the growth of aragonite crystals. Moreover, Cohen and McConnaughey [17] noted that when the calicoblastic ectoderm is elevated from the skeletal surface, it signifies that the coral tissue plays a less direct role in crystal growth. This elevation diminishes contact with the mineralized skeleton, reducing the tissue's influence on CaCO_3_ deposition. Consequently, one may conclude that the growth of aragonite crystals relies more on the environmental conditions within the calcifying space than on the direct involvement of the coral cells, albeit these conditions themselves are tightly controlled by the organism.

Although physicochemical explanations have achieved considerable attention, they do not fully account for key processes such as ionic transportation, organic matrix secretion, and enzymatic activities within calicoblastic cells. While CO_2_ can freely diffuse across cell membranes following its concentration gradient, two ionic species, HCO_3_^−^ and CO_3_^2−^, require specific carriers or proteins to traverse the hydrophobic membrane. In this regard, researchers have identified various ion transporters that facilitate ionic transfer during the mineralization process. In addition to the aforementioned Ca^2+–^ATPase [158], these include Na+/K+ –ATPase (NKA) [159] bicarbonate cotransporters [154, 158], and the Na^+^/Ca^2+^ exchanger (NCX) [160]. This indicates a significant biological involvement in the processes of calcification and mineralization.

Furthermore, the organic matrix plays a crucial role in biomineralization by influencing crystal nucleation and regulating crystal morphology and mineral characteristics at both microscopic and macroscopic length scales [161, 162, 163]. While it is essential for controlling the skeletal building process, the question of whether the organic matrix promotes or inhibits crystal nucleation remains a subject of debate within the physicochemical calcification model [17, 164, 165]. Nonetheless, the documented involvement of the extracellular organic matrix and its significance in a biologically controlled process [29, 166, 167, 168] seem to contradict the purely physicochemical perspective.

### 3.2 Biologically Controlled Coral Calcification

The biologically controlled model of coral calcification posits that the process of biomineralization begins with intracellular vesicles containing calcifying fluid within the calicoblasts (Figure 4b). This model suggests that the process concludes in a biologically regulated extracellular space that also contains calcifying fluid. Similar to the physicochemical model, this extracellular space is situated between the mineralized skeleton and the cell membrane of the calicoderm [109, 169, 170]. Intracellular vesicles are formed by the endocytosis of seawater [64, 144] and typically range in size from approximately 400–600 nm [64]. Calicoblasts concentrate Ca^2+^ in these vesicles through mechanisms such as micropinocytosis [171] or ion exchange via Ca^2+–^ATPase.

Simultaneously, CO_2_ enters the cells from seawater through passive diffusion across the cell membrane, while some is produced via mitochondrial respiration within the cells [154]. A portion of this CO_2_ diffuses into the extracellular calcifying fluid, and another portion is converted to HCO_3_^−^ through a catalytic reaction facilitated by the enzyme carbonic anhydrase [172]. This bicarbonate is then actively transported into the intracellular calcifying fluid vesicles, where it is converted to CO_3_^2−^ at high pH, allowing for efficient binding with Ca^2+^ [64].

Organic molecules produced by calicoblastic cells are released into the extracellular calcifying fluid and injected into the vesicles. As mineral crystals grow, the organic matrix becomes embedded within the developing structure, incorporating itself as the crystals form [65]. It is suggested that the biomineralization process in corals initiates in the regions within the cells that contain calcifying‐related proteins (CARPs), likely responsible for initiating the formation of transient amorphous CaCO_3_ [139]. Eventually, the hydrated amorphous CaCO_3_ nanoparticles created in the intracellular calcifying fluid are released into the extracellular calcifying fluid via exocytosis [173]. In the extracellular calcifying space, these nanoparticles de‐hydrated and transform into amorphous CaCO_3_, and are then deposited onto the growing skeleton and ultimately crystallizes into crystalline CaCO_3_ [65, 144] (Figure 2b). This model emphasizes the role of biological processes in regulating calcification, indicating that coral cells actively participate in forming and transporting mineral precursors for skeleton development.

At this stage, amorphous CaCO_3_ crystallizes through particle attachment, while ions fill the interstitial gaps via ion attachment [65, 144, 174] (Figure 4d). Gilbert et al. [65] and Sun et al. [144] demonstrated that crystallization by particle attachment is the more significant process in terms of generated mass. However, ionic crystallization is inevitable due to the calcifying fluid already being oversaturated with respect to aragonite [65]. CO_2_ diffuses into the calcifying fluid and gets converted into CO_3_^2−^ (Section 3.1) at higher pH, which readily binds Ca^2+^ and consequently fills spaces left by particle attachment [175, 176, 177]. Additionally, the near 100% space filling observed during coral skeleton [65, 144] formation indicates that both mechanisms coexist. It can be inferred that while the mineral structure is primarily formed through particle attachment, crystal growth via a classical ions‐by‐ions mechanism occupy the remaining space to create a compact biomineralized structure [65]. Particle attachment enhances the mechanical properties of biominerals as the amorphous CaCO_3_ nanoparticles crystallize to achieve coherent alignment [178]. Factors such as magnesium content, water availability, organic molecules and confinement conditions influence the stability of amorphous CaCO_3_ and guide its transformation into either calcite or aragonite. These conditions also impact the crystal's chemical composition by affecting the incorporation of trace elements and isotopes [179].

The calicoblastic cells play a crucial role in regulating the transport and secretion of the organic matrix, which may stabilize amorphous CaCO_3_ inside the intracellular vesicles and facilitate its crystallization [174]. Coral skeletons are built by two distinct secretory regions originating from the calicoblastic cells [180, 181, 182]. Centers of calcification at the growing tips indicate the initial stage of mineralization, while the “thickening stage” is driven by the lateral calicoblasts through biochemical secretions. Recent results [132, 183] support the simultaneous formation of various skeletal structures at different locations within the coral skeleton, reinforcing the compartmentalized model. Their findings indicate that coral growth is a complex and dynamic process, with multiple skeletal components developing concurrently.

### 3.3 Biologically vs. Physicochemically Controlled Coral Calcification

Reconsidering these two models, it appears that the interplay between physicochemical and biological mechanisms is essential for coral calcification. Obviously, the coral polyp tightly controls the biomineralization process by generating precursors, controlling the biochemical environment, and supplying the mineralization site. The generation of mineralization pockets through lifting tissue away from the mineralized skeleton generates a space akin to a reaction chamber in which physicochemical processes can take place. However, organic macromolecules supplied by the coral polyp are also crucial here. Each biomineralization model contributes valuable insights, but neither fully captures the entire calcification process. Quantification of their individual contributions is a challenge as their in vivo signatures are difficult to differentiate.

We believe that the information gathered here supports an interpretation that both processes really run concurrently as part of the same biomineralization process. The animal modifies seawater to create an extracellular calcifying fluid, influencing pH and Ca^2+^ transport, as proposed by the physicochemical model. However, this is done by calicoblastic cells and biomineralization‐related proteins regulate crystal formation. There seems to be no reason that the two viewpoints are mutually exclusive. In fact, coral biomineralization shows aspects of both models but the tight biological control and the crucial role of organic matter favor a hybrid biologically controlled coral calcification that employs physicochemical mechanisms (Section 4).

## 4 Structure Building Process

The intricate nature and spatial variability of the skeletal development process across different genera highlight the complex biomineralization mechanisms involved in corals [184]. Overall, the structure‐building process is explained by two different models:

- The two‐step mineralization model as proposed by Cuif and Dauphin [182] and Cuif et al. [128, 181] assumes that skeletal formation occurs in two distinct phases. The model emphasizes differences in the timing of the formation of CoC and aragonite needles. It introduces the concept of an “early mineralization zone” for the CoC.
- The layered model as proposed by Stolarski [48] suggests that coral skeletons are built by sequential deposition of thin, organic‐rich layers (i.e. CoCs) where the mineralization process occurs. This results in a septal skeleton consisting of superimposed layers of organic material‐enriched and mineral phases. The model assumes that continuity exists between CoC and aragonite needles, which makes the distinctions between these structures less clear but it means that successive layers of CoC‐aragonite‐CoC‐aragonite are formed. Comparable models have also been investigated across skeletal structures of other organisms [185, 186].

Both structure building models emphasize calcification processes that are tightly controlled by calicoblasts, reinforcing the applicability of the biologically controlled calcification model. Furthermore, in vitro studies reveal that the composition of the organic matrix and the skeletal structure synthesized by polyps grown through tissue culture techniques closely resemble that of the parent coral colony. This resemblance highlights the significant role of genetic factors in determining skeletal morphology and characteristics within a species [187].

Over the past decade, more than a hundred extracellular matrix proteins have been identified, which are secreted into the calcification space by calicoblasts [188, 189]. These include CARPs, von Willebrand factor type D (vWFD) domain‐containing proteins, fibronectin, alpha‐collagen type I and uncharacterized skeletal proteins [29, 188]. These proteins control the crystal formation process in extracellular calcifying fluid [190] (Section 3). Mass et al. [191] studied the expression patterns of various proteins during the early developmental stages of stony corals. CARP1, a member of the extracellular matrix proteins, is localized adjacent to newly formed CaCO_3_ particles, while CARP2 promotes aragonite formation. CARP4, CARP5, and CARP6 play key roles in calcium binding during the biomineralization process [191], whereas CARP3 facilitates CaCO_3_ crystallization toward Mg‐calcite [31, 192]. This observation suggests that not all CARPs are involved in the selection of biomineral morphology [192]. Additionally, corals modulate the expression of CARP‐related genes to regulate the production of these proteins during the mineralization process [15]. Due to their abundance of carboxyl groups, CARPs are among the most prevalent and efficient cation‐binding agents available to corals. These substances facilitate the binding of mineral precursors and serve as a form of “glue” in biomineralization processes [28].

Von Euw et al. [56] observed that CaCO_3_ crystals arise from ≈3 µm thick organic fibre‐like material located perpendicular to the plane of aragonite crystal growth in CoCs [56] (Figure 3). “Immature” aragonite appears as nanometre sized particles embedded in the surface of the organic fibre as detected through Raman spectroscopy. These findings indicate that the skeletal organic matrix found in CoCs forms an organic foundation that facilitates the nucleation of the mineral phase [139]. An interesting question in this context is, where is the next CoC and why?

**FIGURE 5:**
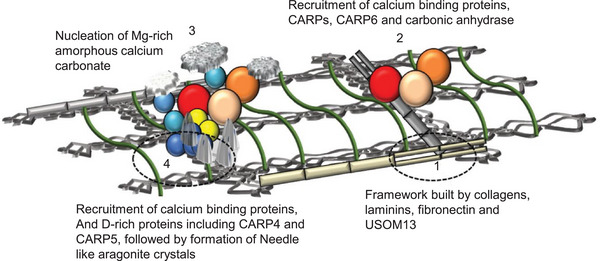
A model of biologically regulated mineralization in corals: The proposed model illustrates how needle‐like crystals form on a network of organic matrix. Image is adopted under terms of the CC‐BY license from Mummadisetti et al. [188].

Mummadisetti et al. [188] proposed that skeletal vWF domain‐containing proteins, along with collagen, laminin, and uncharacterized skeleton‐organizing matrix protein (USOMP13) form a structural framework (Figure 5). In this framework, CARP2 and CARP6 (rich in glutamic acid) attach to collagen in the presence of carbonic anhydrase, initiating the formation of Mg‐rich amorphous CaCO_3_. Subsequently, CARP4, CARP5 (rich in aspartic acid proteins), and other binding proteins are recruited to this framework. Ultimately, this leads to the formation of amorphous CaCO_3_ and eventually to crystallization into needle‐like aragonite [188, 192].

Numerous recent studies [3, 38, 193, 194, 195] have demonstrated that organic molecules are found both within and around the aragonite needles. Atomic force microscopy data indicates that the acicular aragonite needles are primarily made‐up of nanograins of a typical size of approximately 20 − 100 nm (Figure 1a), which are coated with organic material [3, 38, 193, 194]. In an in vitro crystallization experiment, Falini et al. [38] observed the aggregation of CaCO_3_ nanoparticles forming rods and dumbbells in the presence of organic matrices extracted from various coral species, while rhombohedral CaCO_3_ crystals were formed in the absence of the organic matrix. These studies [38, 193, 194] suggest that organic molecules not only provide nucleation sites for the initial CaCO_3_ deposition but also play a role as modifiers in the crystallization process, influencing both the shape and growth direction of aragonite crystals.

In the beginning of coral skeletogenesis amorphous CaCO_3_ precursors containing Mg‐calcite appear in the form of rods (approximately 1–5 µm in size) and/or dumbbell‐like structures (approximately 10–35 µm in size), depending upon species at initial sites of calcification which correspond to CoC [15, 45, 119] (Figure 3a). Neder et al. [15] reported that those structures are composed of nanogranular particles ranging from 50–100 nm in size, a feature that resonates with the nanogranular setup of aragonite needles. Aragonite needles then grow outward from these dumbbell‐like precursors with homogeneous crystallographic orientations. Sugiura et al. [196] demonstrated that the center of calcification is biologically constructed, while the subsequent thickening of the skeleton by fibrous aragonite nanorods occurs through physicochemical processes, producing patterns closely matching crystal growth observed in laboratory systems. During crystal growth (Figure 3b), the organic matrix surrounding the nanogranules become occluded within the mineral crystal [65]. The necessary mineral ions are transported to the mineralizing surface either through active transport by calicoblasts [197] or through direct exchange of the calcifying fluid with seawater and/or by diffusion from surrounding seawater [198, 199] (see also Section 4 and Figure 2). Mg^2+^ ions also play an important role in the selection of the CaCO_3_ polymorph. In vitro studies suggest that a magnesium to calcium ratio similar to that found in seawater (Mg: Ca ≈4.40–6.40) [200] promotes the crystal growth toward aragonite [38].

In conclusion, the local building process of and around one polyp begins within the formation of CoCs, where organic molecules and mineral granules accumulate. Acicular aragonite needles grow outward from the CoC and form bulk structures such as sclerodermites, which ultimately make up the coral skeleton. Throughout this process, various extracellular matrix proteins, such as CARPs, facilitate the binding of mineral precursors and control crystal formation. Based on the evidence presented, we believe that the layered model is more favorable as it encapsulates the dynamic interactions between minerals and the organic matrix, reflecting the continuous nature of skeletal development. This model better explains the intricate mechanisms of biomineralization in corals, showcasing the interplay between genetic control, biochemical processes, and environmental influences in the formation of robust skeletal structures. It also reinforces the notion of a biologically controlled coral calcification process that employs physicochemical mechanisms (see also Section 3).

## 5 Coral's Skeletogenesis Process—Parallelized, Multiscale Biofabrication

There seems to be a gap in the literature between understanding the biomineralization process in corals per se and the formation of bulk skeletal material, i.e., the scaled “manufacturing” of skeletal material. It is clear that the former enables the latter. However, viewed from a (bio)materials science perspective, understanding bulk material generation with comparable material properties in vast quantities is as important as understanding biomineralization itself as it facilitates scaling and offers a view into an interesting manufacturing framework.

**FIGURE 6:**
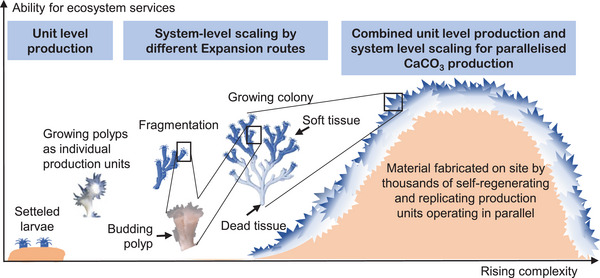
Skeletal construction and expansion process—parallelized, multiscale biofabrication: Complexity arises from the collective action of thousands of self‐regenerating and replicating polyps which we understand as the production unit. Utilizing multiple ways to expand, this generates a self‐configuring, redundant system to produce complex reefs. The adaptive modular structure together with decentralized decision making and decentralized control generates an efficient biomanufacturing system. The distribution of individual polyps furthermore enables this ecosystem to realize distributed sensing for chemicals, pressure and pH—even including unit‐death as a feedback system so that reaction to external stimuli is inbuilt. Finally, this system is inherently circular generating skeletal materials that are stiff and strong.

So let us step back for a moment and reconsider the generation and expansion of the ecosystem in light of this manufacturing framework (Figure 6). Generally, each polyp functions as a specialized production unit in a larger body, i.e., the reef. They are capable of operating autonomously (up to a certain point) but guided by an overall goal, i.e. access to energy sources that in turn drive ecosystem engineering [73]. There is no “chief engineer” guiding production. Rather the collective of polyps automatically and independently execute manufacturing tasks like a decentralized swarm of bioprinters. The material is fabricated on‐site to enable the growth and reconfiguration of modular structures—from individual polyps to branches, colonies, and full reefs—much like a fusion of coral polyps and 3D printing technology.

While fabricating, the individual polyps are able to “diagnose” their environment [201, 202, 203, 204] and adapt to subtle environmental cues [94, 95] (to the point where they are overwhelmed by multiple stressors [12]). This decentralized coordination can result in a reversible growth arrest that preserves mechanical integrity of the skeleton [205]. Such coordination can span disconnected tissues and even neighboring colonies, mediated by mechanical coupling through the skeleton [206] and biocommunication via environmental and chemical cues [207, 208]. At the same time, individual polyps or regions of a coral colony can respond differently to the same stressor, owing to variation in physiological states and in the colony's internal transport and signaling networks [209, 210]. Moreover, while polyps look like they are freelancing, their output is coordinated by overarching governing principles [73]. Consequently, the complexity in the ecosystem engineering does not come from a central plan but from the interactions of thousands of simple polyps—which resembles aspects of swarm action.

In addition to that, these polyps employ modular growth and expansion schemes in which individual polyps are capable to divide and grow in situ to multiply if needed. Four mechanisms on three different length scales seem to facilitate modular growth and expansion and, thus, scaling of the biomanufacturing process in generating the mineralized skeleton (Figures 4 and 6): (i) continued polyp growth (microscale, controlled by calicoblasts); (ii) polyp tissue retraction (mesoscale, tissue motion); (iii) budding (macroscale, whole polyps, i.e., “units” are added); and (iv) fragmentation (macroscale, whole colonial compartments).

Polyps continue to grow in size and secrete CaCO_3_, acting as local manufacturing units, resulting in the addition of new layers to the existing structure (Section 3). This subsequent layering creates a thickened skeletal structure over time, allowing the skeleton to expand both radially and longitudinally. Physicochemical models suggest that growth layers are influenced by environmental conditions [70], while biological models emphasize that layering is affected by various factors, including the organic matrix that guides the entire mineralization process [40]. Polyps create free space for continued aragonite crystallization through uplifting [138, 156, 211, 212], i.e. movement of the “3D printing unit”.

Polyp tissue retraction plays a crucial role in skeletal expansion. In their study on the CWC *Caryophyllia huinayensis*, Heran et al. [99] observed that as the basal plate and septa thickened and the U‐shaped columella protruded from the center, the soft‐tissue—consisting of ectoderm and endoderm layers—retracted from the basal plate, leaving the base exposed. This exposed area was covered again after 150 days of growth and exposed once more approximately 895 days later. This underlines the adaptability of the individual manufacturing unit in the creation of skeletal mass.

Budding resembles a modular skeletal growth and expansion strategy that efficiently increases the number of on‐site manufacturing units. New polyps form from existing ones, contributing to the enlargement of the skeleton (Figure 6), potentially with distinct structural features such as distances, diameters, and other sizes, as exemplified by Peña Fernández et al. for *L. pertusa* [54]. Nevertheless, this mechanism is in so far an interesting manufacturing concept as the individual manufacturing unit replicates itself to scale production—a concept that current (bio)fabrication methods do not yet deliver and which would constitute a step change in additive manufacturing.

In colony fragmentation, a piece of tissue with or without mineralized skeleton is separated from the colony. Once the separated piece comes in contact with a suitable surface it fuses with it and grows into a separate colony employing mechanisms (i‐iii) as described above [107, 108]. While this allows for colonization of potentially new territory, from a technological perspective, this amounts to having a fully functional, parallelized manufacturing platform with all features described above (Figure 6) ready to be moved to a new location away from its point of origin. The system therefore becomes conveyable fully functional which ultimately draws from the bioengineering capability of a parallelized, multiscale manufacturing platform, where hundreds of thousands of individual units produce large quantities of material with consistent properties.

## 6 How to Build Like Corals—A Perspective

Corals routinely achieve what modern manufacturing still struggles with: scalable, decentralized, self‐organized, multiscale production of stiff and strong materials with scarce resources in terms of energy and precursor materials. Translating these natural advantages into biomedical, environmental, and industrial applications requires precise process control to maximize material yield and scale up production. At the same time, these processes must fine‐tune bio‐physicochemical attributes such as material architecture, controlled porosity, interconnectivity, mineral phase, and crystallographic texture to achieve properties similar to their natural antetypes. Existing manufacturing processes are inadequate to achieve this. Moreover, there are no self‐regenerating and replicating additive manufacturing systems that could realize the observed adaptability. However, there are promising developments that may enable all this.

**FIGURE 7:**
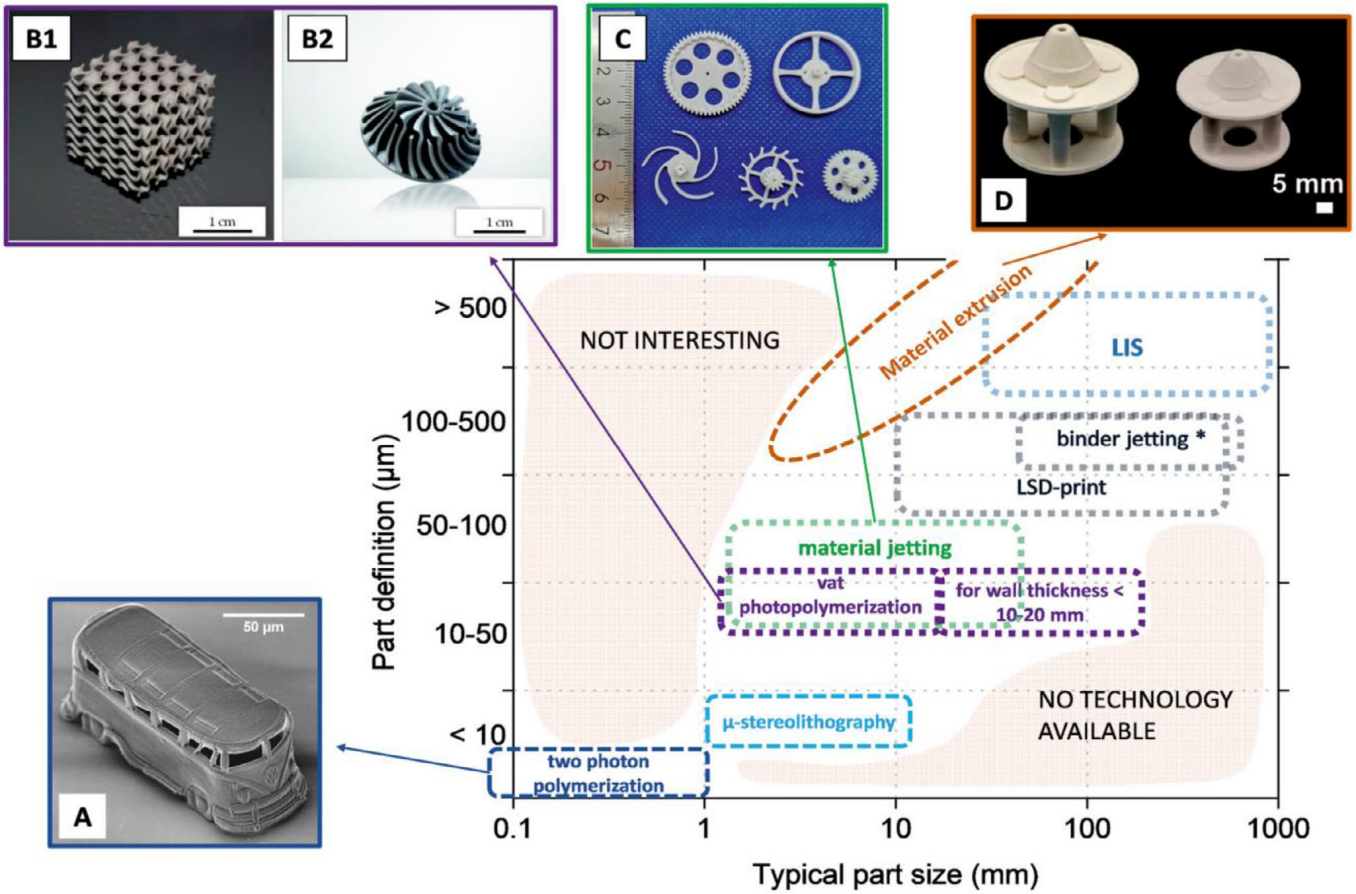
Additive manufacturing technologies for dense advanced ceramics illustrated along their typical part size and the achievable part definition. (A) shows a zirconia structure made by two photon polymerization. (B1 & 2) illustrates silicon nitride parts made by vat photopolymerization, specifically lithography‐based ceramic manufacturing. (C) shows zirconia parts made by material jetting, specifically nanoparticle jetting. (D) Structures made by materials extrusion, specifically infrared‐assisted direct ink writing. Corals appear to be capable to manufacture across the part sizes as well as the part definition illustrated here but with one “technology”. Given their biomineralization and structural building processes (Sections 3 and 4), they cover the diagonal of available technologies (white) but could facilitate to tap into the area of unavailable technologies (bottom right). Image reproduced under terms of the CC‐BY license from Zocca and Günster [215].

First of all, manufacturing systems exist both for metals as well as for ceramics [213, 214, 215] that are capable to bridge the necessary length scales (Figure 7). Second, by leveraging the potential of additive manufacturing in crafting multiscale structures, researchers are engineering synthetic architectures that strive to mirror the defining features of corals. For example, Morais et al. [216] used vat photopolymerization to 3D print bulk CaCO_3_ with complex macroscopic geometries using a low‐viscosity PEGDA‐based resin containing 35 vol% CaCO_3_. The method produced coral‐like structures, yet challenges remain in achieving comparable mechanical properties, mitigating cracking, and realizing multiscale manufacturing across relevant length‐scales as coral polyps do. Producing CaCO_3_ constructs, however, is not straightforward, as several key issues such as formulating a printable suspension, sintering CaCO_3_ without thermal decomposition and fabricating complex geometries with multiscale porosity [217] remain. Thermal decomposition can be minimized by employing a two‐step thermal cycle, consisting of debinding below 500°C in air and sintering at 850°C [216]. However, shrinkage and low mechanical properties remain an issue.

Organic copolymer–preceramic inks enable block copolymer self‐assembly [218] to delivers polyp‑like nanoscale‑to‑mesoscale structuring and hierarchical porosity. Direct ink writing (DIW) with a thiol‑crosslinked PCS/PMMA‑b‑PnBA‑b‑PMMA ink, followed by UV curing locks the nanostructure. Subsequent inert pyrolysis (∼800°C) converts polycarbosilane (PCS) to silicon oxycarbide (SiOC) while removing the triblock copolymer porogen, leaving nanocoral morphology [219]. Moreover, ice‑templating (freeze‑casting) enables control over pore size and architecture, and represents a versatile route for biomineral‑inspired structures, including nacre‑like materials [220]. In this framework, block copolymer self‐assembly defines nanoscale features (∼10–100 nm) [218], while additive manufacturing techniques, such as DIW and photopolymerization (digital light processing or stereolithography) prescribes mesoscale‐to‐macroscale architecture with feature size from ∼10 to 500 µm and overall build size from millimeters to centimeters [215, 219, 221]. Thus, bridging length scales from crystal assembly (Figure 4) to bulk material generation.

Despite these advances, synthetic materials continue to face limitations in mechanical strength, inconsistent properties, scalability, and reproducibility. In contrast, corals and in particular cold‐water corals mineralize dense CaCO_3_ under challenging conditions [9, 44, 222] by first depositing hydrated amorphous precursors, which later transform and densify [64]. Hu et al. [223] described a cold‑sintering method in which careful control of water content, temperature, and pressure converts amorphous CaCO_3_ into hydrated crystalline intermediates such as ikaite and monohydrocalcite (MHC). These intermediates were then converted into dense aragonite ceramics at 80°C in the presence of Mg^2+^. Materials derived from MHC showed excellent mechanical performance, with hardness 4.0 GPa, Young's modulus 70 GPa, and flexural strength 55 MPa, values comparable to those of natural coral skeletons. Although this route offers a more sustainable, energy‑efficient path to high‑performance ceramics, challenges remain in controlling phase stability, limiting defects, and reliance on expensive equipment that impedes industrialization (Figure 7). Integrating cold sintering with spatially selective crystallization could enable hierarchically structured, compositionally graded CaCO_3_ based materials with controlled grain boundaries and improved mechanical and functional properties [224]. Corals not only densify precursors but also set crystallographic orientation with precision. In a synthetic analogue [20], “Writing crystallography”, laser‐induced, spatially controlled crystallization of Mg‐stabilized amorphous CaCO_3_, creates multiple crystalline phases at high spatial resolution, enabling mechanistic insight and local control of crystallography [214]. Recent techniques that prolong the stability of amorphous CaCO_3_ by adding biomolecules (i.e., collagen) [225] have opened new possibilities for fabricating complex, hierarchical structures at low temperatures in environmentally friendly ways, greatly increasing design flexibility. The main challenge now is to achieve truly significant gains in strength and toughness, which will require advancements in interface control, material selection, and processing methods [225].

Replicating coral minerals is difficult because biomineralization depends on dynamic, multicomponent processes rather than simple chemistry. Researchers are using microbes [226] and engineered cells [227] to biomineralize coral‐like CaCO_3_, creating “living building materials” [226, 227] that can grow and self‐heal with the help of synthetic biology and scaffolds [228, 229, 230]. So far current systems are limited by slow, small‐scale mineralization, insufficient material complexity and mechanics, plus challenges in cell viability, morphogenesis control and biosafety concerns [231, 232]. Consequently, these materials remain experimental and not ready for large‐scale material construction, requiring further interdisciplinary research [231].

Emerging methods, as outlined above (also summarized in Table 1), form a coral‐inspired manufacturing stack encompassing macro‐architecture, hierarchical porosity, nanoscale order, low‐temperature precursor control and local crystallography. The main challenges include material compatibility and mechanical performance, control of multiscale coupling and stress management, scalable throughput at acceptable energy cost, reliable operation in wet/ionic media and ensuring biosecurity and long‐term stability. The central opportunity is to fuse these capabilities into a unified, adaptive, self‐sustaining platform that approaches the efficiency, scalability, and autonomy of coral biomineralization.

**TABLE 1:** Overview of technologies recently used for fabricating coral‐like materials. We summarize advanced synthesis processes for CaCO_3_‐based materials, along with their outcomes and associated challenges. Images reproduced with permission.

Fabrication strategy	Process schematic	Fabricated material	Principal outcomes	Major Challenges	Refs.
Vat‐Photopolymerization	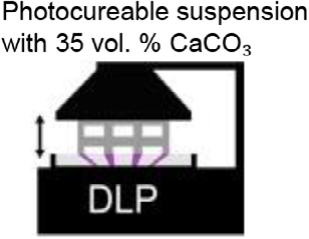	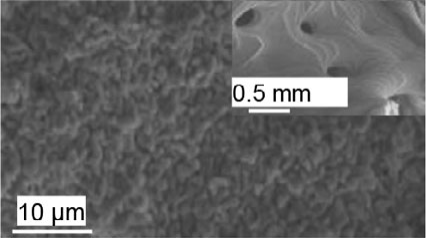	Macroscale coral like parts	Material cracking, limited mechanical strength	[216]
Direct Ink Writing	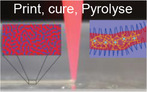	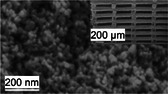	Nanocoral morphology with multiscale porosity	Limited mechanical strength, inconsistent thermal properties, scalability issues	[219]
Cold Sintering	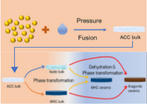	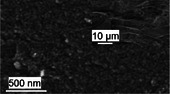	Biomimetic material fabrication with excellent mechanical properties	High pressure, scalability, material defects durability check,	[223]
Bio‐inspired 3D Printing	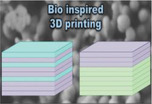	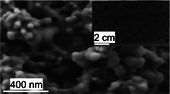	Using biomolecules in printing, long term stability, Low temperature processing	Defects at layers interfaces, Limited mechanical enhancement	[225]
Writing Crystallography (laser induced)	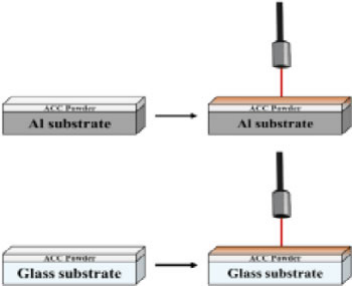	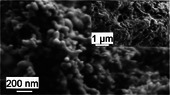	Multiple phase control, High spatial resolution and 3D capability	Scalability, substrate dependant outcome, bulk processing	[214]
Living Building Materials	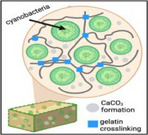	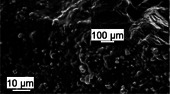	Actively fix carbon dioxide, biomineralization, self‐healing	Slow and small‐scale mineralization, insufficient material complexity, biosafety	[227]
Coral‐mimetic System	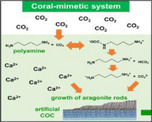	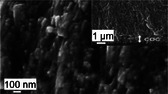	Bioinspired efficiency, polymorph control, nanostructure tunability	Slow CO2 capture rate, limited continues production, Optimization complexity	[233]

A more practical strategy is process‑level biomimicry, a shift from compositional mimicry toward process‐level biomimicry offers a more tractable strategy. To achieve this complete biomineralization toolkit should include tailored organic additives (polyamines, peptides, or matrix analogues) to modulate nucleation and crystal habit, precise ionic delivery, a control on local carbonate chemistry. A coral‑inspired method used biogenic polyamines to capture atmospheric CO_2_ and to direct nucleation and crystal growth, producing aragonite nanorod films that closely replicate coral aragonite morphology [233]. By combining CO_2_ capture from the provided source with controlled organic templating, this strategy leveraged natural biomineralization pathways to selectively tune CaCO_3_ polymorphism and microstructure using a scalable setup. Embedding such approaches into continuous, fed‑batch, or microfluidic reactor platforms, and coupling them with real‑time monitoring and feedback loops, can enable systematic mapping of robust processing windows and improve reproducibility. Microfluidic platforms are uniquely suited to elucidate and control calcium carbonate formation and to evaluate the biomineralization factors [234]. The gradient microfluidic device enabled precise variation of Mg^2^^+^ concentrations, which were used to investigate CaCO_3_ polymorph selection [235]. Such systems allow fine control over supersaturation and additive delivery, providing mechanistic insight into biomineralization and practical routes to produce tailored CaCO_3_ materials with defined architectures.

Table 1 summarizes advanced processes developed for the synthesis of coral like materials (CaCO_3_), along with their outcomes and associated challenges. While various techniques are available for the fabrication of artificial reefs, these fall outside the scope of this review and are therefore not included in this section or table.

Different multimaterial bioprinting strategies are being explored to mimic coral like soft tissues, combining bio inks such as gelatin methacrylate (GelMA) [91] or alginate methacrylate (AlgMa) [236], mixed with poly(ethylene glycol) diacrylate (PEGDA) [91, 236]. This type of 3D bio printed coral structures can be combined with engineered microbial consortia embedded within hydrogels for functions like sensing or self‑healing and can feature with integrated chemical channels for mass transport. Coral itself functions much like a natural soft robot: each polyp is a flexible sensing unit, responsive to light, water flow, and chemical signals in its environment. Inspired by this, 3D printed soft bodies can be fabricated with embedded microfluidic networks [237] that create programmable droplet microreactors [238, 239], similar to intracellular vesicles in corals, enabling calcium carbonate precipitation with spatial and temporal control [240]. Moreover, instead of relying on living cells, biomineralization proteins [241], specifically designed short or ultrashort peptides [242, 243], or even individual amino acids [244, 245] can be introduced into these droplets to regulate the mineral formation process. The controlled deposition of mineralized droplets onto surfaces could enable the growth of rigid, coral‐like structures over time. Using (droplet) microfluidics offers programmable operations, tuneable nucleation by controlling supersaturation and scalable parallelization [246].

Additionally, sensors based on carbon nanotubes for detecting proteins [247, 248] and lipids [249], as well as optical nanothermometers [250, 251] and nanoprobes for monitoring biotic and abiotic factors [252, 253] have already been used in related studies. These technologies could be integrated to create a real‐time sensing and feedback loop within these systems [87]. Working individually or as a swarm, such robots could enable programmable, scalable mineralization strategies that bridge biological inspiration and engineered systems.

The techniques reviewed in Section 6 are promising as they bridge necessary length scales. They illustrate avenues of inquiry that may enable us to advance the practical application of the knowledge gathered here and catalyze the development of next‐generation materials technologies. However, parallelization and the ability to adapt can only be trivially achieved with current technological solutions by (re‐)assembling existing manufacturing units. In contrast, coral systems parallelize and adapt on demand by self‐replication, decentralization, self‐regulation, and redundancy (Section 5). Thus, they increasing the number of manufacturing units as needed or reducing this capability if resources become scarce. This organic scaling capacity is at the moment not technologically possible.

## 7 Conclusion

We here aimed at synthesizing how corals construct their skeletal material and develop significant structures with reproducible properties over relatively short time scales so that we could then recast coral growth as a multiscale, parallelized biofabrication process that can catalyze the development of next‐generation materials technologies. To address this, we provided a brief overview of the life cycle and anatomy of corals; we explored the biotic and abiotic pathways involved in mineralization, focusing on potential interactions between organic matrices and CaCO_3_; we examined the mechanisms of bulk skeletal material production at macroscopic length scales; and we looked at how these processes interconnect to form a multiscale, parallelized biotechnological framework for manufacturing these biomaterials.

We enriched these analyses with a perspective on existing techniques how they may enable us to recreate the multiscale, adaptive, parallelized biofabrication techniques used by corals. However, if we are to mimic these manufacturing methods, it is imperative to look beyond the individual calcification unit (cellular system and individual polyps) and also take into account their decentralized, self‐regulated, and coordinated action. The processes of biomineralization in corals are intricate and interdependent, encompassing a range of mechanisms that collaboratively enhance coral growth and adaptation. Some of these mechanisms go significantly beyond the current state‐of‐technology in manufacturing but represent interesting concepts to move the field. Consequently, harnessing this interconnected multiscale, parallelized biotechnological framework for manufacturing CaCO_3_ and other (bio)materials could yield significant advancements in biomimetic designs and sustainable approaches to material engineering.

## Author Contributions

**Asma Rehman**: Investigation, Data Curation, Visualization, Writing – Original Draft. **Uwe Wolfram**: Conceptualization, Supervision, Project administration, Funding acquisition, Writing – Original Draft. **Marta Peña Fernández**, **Kristina Beck**, **Sebastian Hennige**, **Gavin Foster**: Writing – Review and Editing.

## Conflicts of Interest

The authors declare no conflict of interest.

## Data Availability

The authors have nothing to report.
